# Myocarditis in anti-synthetase syndrome: clinical features and diagnostic modalities

**DOI:** 10.1093/rheumatology/kead541

**Published:** 2023-10-05

**Authors:** Giacomo De Luca, Corrado Campochiaro, Anna Palmisano, Elisa Bruno, Davide Vignale, Giovanni Peretto, Simone Sala, Arianna Ferlito, Maria Bernardette Cilona, Antonio Esposito, Marco Matucci-Cerinic, Lorenzo Dagna

**Affiliations:** Unit of Immunology, Rheumatology, Allergy and Rare Diseases, IRCCS San Raffaele Hospital, Milan, Italy; School of Medicine, Vita-Salute San Raffaele University, Milan, Italy; Unit of Immunology, Rheumatology, Allergy and Rare Diseases, IRCCS San Raffaele Hospital, Milan, Italy; School of Medicine, Vita-Salute San Raffaele University, Milan, Italy; School of Medicine, Vita-Salute San Raffaele University, Milan, Italy; Clinical and Experimental Radiology Unit, Experimental Imaging Center, IRCCS San Raffaele Hospital, Milan, Italy; School of Medicine, Vita-Salute San Raffaele University, Milan, Italy; Clinical and Experimental Radiology Unit, Experimental Imaging Center, IRCCS San Raffaele Hospital, Milan, Italy; School of Medicine, Vita-Salute San Raffaele University, Milan, Italy; Clinical and Experimental Radiology Unit, Experimental Imaging Center, IRCCS San Raffaele Hospital, Milan, Italy; Department of Cardiac Electrophysiology and Arrhythmology, IRCCS San Raffaele Hospital and University, Milan, Italy; Department of Cardiac Electrophysiology and Arrhythmology, IRCCS San Raffaele Hospital and University, Milan, Italy; Unit of Immunology, Rheumatology, Allergy and Rare Diseases, IRCCS San Raffaele Hospital, Milan, Italy; School of Medicine, Vita-Salute San Raffaele University, Milan, Italy; Unit of Immunology, Rheumatology, Allergy and Rare Diseases, IRCCS San Raffaele Hospital, Milan, Italy; School of Medicine, Vita-Salute San Raffaele University, Milan, Italy; School of Medicine, Vita-Salute San Raffaele University, Milan, Italy; Clinical and Experimental Radiology Unit, Experimental Imaging Center, IRCCS San Raffaele Hospital, Milan, Italy; Unit of Immunology, Rheumatology, Allergy and Rare Diseases, IRCCS San Raffaele Hospital, Milan, Italy; Department of Experimental and Clinical Medicine, University of Florence, and Division of Rheumatology AOUC, Florence, Italy; Unit of Immunology, Rheumatology, Allergy and Rare Diseases, IRCCS San Raffaele Hospital, Milan, Italy; School of Medicine, Vita-Salute San Raffaele University, Milan, Italy

**Keywords:** anti-synthetase syndrome, myocarditis, cardiac magnetic resonance

## Abstract

**Objectives:**

Myocarditis is an overlooked manifestation of anti-synthetase syndrome (ASS). Our study describes the clinical and instrumental features of ASS myocarditis and evaluates the performance of cardiac MRI (CMRI) with mapping techniques in assisting diagnosis of ASS myocarditis.

**Methods:**

Data from patients with ASS were retrospectively analysed. CMRI data for patients diagnosed with myocarditis, including late gadolinium enhancement (LGE), T2 ratio, T1 mapping, extracellular volume (ECV) and T2 mapping, were reviewed. Myocarditis was defined by the presence of symptoms and/or signs suggestive for heart involvement, including increased high-sensitive troponin T (hs-TnT) and/or N-terminal pro-brain natriuretic peptide (NT-proBNP), and at least an instrumental abnormality. The clinical features of patients with ASS with and without myocarditis were compared. A *P*-value of <0.05 was considered statistically significant.

**Results:**

Among a cohort of 43 patients with ASS [median age 58 (48.0–66.0) years; females 74.4%; anti-Jo1 53.5%], 13 (30%) were diagnosed with myocarditis. In 54% of those 13 patients, myocarditis was diagnosed at clinical onset. All patients with ASS with myocarditis had at least one CMRI abnormality: increased ECV in all cases, presence of LGE in 91%, and increased T1 and T2 mapping in 91%. The 2009 Lake Louise criteria (LLC) were satisfied by 6 patients, and the 2018 LLC by 10 patients. With the updated LLC, the sensitivity for myocarditis improved from 54.6% to 91.0%. Patients with ASS with myocarditis were more frequently males (53% *vs* 13%; *P* = 0.009) with fever (69% *vs* 17%; *P* = 0.001), and had higher hs-TnT [88.0 (23.55–311.5) *vs* 9.80 (5.0–23.0) ng/l; *P* < 0.001], NT-proBNP [525.5 (243.5–1575.25) *vs* 59.0 (32.0–165.5; *P* = 0.013) pg/ml; *P* = 0.013] and CRP [7.0 (1.7–15.75) *vs* 1.85 (0.5–2.86) mg/l; *P* = 0.011] compared with those without myocarditis.

**Conclusion:**

In ASS, myocarditis is frequent, even at clinical onset. Patients with ASS with myocarditis frequently presented with fever and increased CRP, suggesting the existence of an inflammatory phenotype. The use of novel CMRI mapping techniques may increase diagnostic sensitivity for myocarditis in ASS.

Rheumatology key messages

- Myocarditis is frequent in anti-synthetase syndrome (ASS), even at clinical onset, and is associated with peculiar clinical features.
- Detection of myocarditis in ASS even at the subclinical stage is crucial to allow prompt therapeutic intervention.
- Cardiac MRI mapping techniques can improve our ability to detect myocardial inflammation in patients with ASS with myocarditis.

## Introduction

Anti-synthetase syndrome (ASS) is a rare immune-mediated systemic disease that belongs to the idiopathic inflammatory myopathies (IIMs). ASS is characterized by the presence of any one of a number of mutually exclusive autoantibodies directed against amino-acyl transfer RNA-synthetases [1]. The syndrome has a broad spectrum of clinical features, including interstitial lung disease (ILD), myositis, arthritis/arthralgia, RP, fever, mechanic’s hands, and skin rashes [1]. In patients with ASS, myocarditis is seldom diagnosed [2]. In patients with autoimmune diseases, the diagnosis of myocarditis is a challenge. This is due to the clinical presentation, which is frequently subclinical and not specific, and the absence of a definite diagnostic algorithm [3–6]. An early diagnosis of myocarditis is essential for prompt commencement of immunosuppressive therapy, avoiding early casualties and late-stage cardiac complications. Thus, in patients with ASS, a comprehensive characterization of clinical and instrumental features is crucial for the early and even subclinical recognition of myocarditis.

Cardiac MRI (CMRI) is the tool of choice for the non-invasive diagnosis of myocarditis and for the morpho-functional and structural characterization of the myocardium [7–9]. It is widely used for diagnosis and monitoring inflammatory cardiomyopathies and for the characterization of myocardial inflammation and myocardial fibrosis [7–11]. The presence of at least two out of three ‘Lake Louise criteria‘ (LLC) is required for the CMRI diagnosis of myocarditis [8]. However, in CTDs traditional CMRI has limitations in detecting myocarditis, and there is a risk of false-negative results in patients with coexisting myositis [12]. Novel CMRI techniques, which include T1 mapping, T2 mapping, and extracellular volume (ECV) quantification, can, however, be incorporated into the revised LLC and may overcome these limitations [8].

The aims of the present study were: (1) to comprehensively describe the clinical and instrumental features of ASS myocarditis, (2) to investigate the clinical features associated with myocarditis in patients with ASS, and (3) to investigate the performance of CMRI with mapping techniques in detecting myocardial inflammation.

## Methods

### Patients

Data from patients diagnosed with ASS according to Solomon’s criteria [13] and followed at the IRCCS San Raffaele Hospital (Milan, Italy) between December 2016 and December 2022 were retrospectively reviewed.

The study is in agreement with the recommendations of Declaration of Helsinki. The Institutional Review Board at San Raffaele Hospital specifically approved the study, and written informed consent to participation was obtained from each patient.

All enrolled patients underwent a comprehensive evaluation of their disease characteristics, including: disease onset and duration, autoantibody profile, inflammatory markers (ESR, CRP), presence of skeletal myositis, arthritis, fever, ILD and/or skin rashes, and traditional cardiovascular risk factors. All patients with ASS underwent the same evaluation, which is described in the Supplementary Material, available at *Rheumatology* online.

ASS with myocarditis was defined by ASS plus the presence of symptoms and/or signs of cardiac involvement (dyspnea, palpitations, chest pain, signs of congestive heart failure) associated with increased serum levels of high-sensitivity troponin T (hs-TnT) and/or N-terminal pro-brain natriuretic peptide (NT-proBNP) and at least an instrumental sign of cardiac involvement from the 24 h-ECG holter and/or echocardiography and/or CMRI.

The predominant signs or symptoms that led to the suspicion of myocarditis were considered for the definition of the clinical presentation pattern (see Supplementary Material, available at *Rheumatology* online) [14].

All patients with ASS diagnosed with myocarditis, as previously defined, underwent a further comprehensive non-invasive cardiologic evaluation with CMRI with mapping techniques. CMRI was performed on 1.5-T systems (Achieva dStream, Philips Medical Systems, Eindhoven, The Netherlands) equipped with a 32-channel phased-array coil [7, 11]. CMRI analysis was performed as previously described [7, 11] and included: volume and function, early and late gadolinium enhancement (EGE and LGE, respectively), T2-weighted Short-tau Inversion Recovery(STIR) oedema-sensitive images, T1 mapping*,* T2 mapping and ECV (see Supplementary Material, available at *Rheumatology* online).

In selected cases, an endomyocardial biopsy (EMB) was performed, according to current guidelines [5, 6, 15–20], to confirm the diagnosis and to rule out a viral aetiology (see Supplementary Material, available at *Rheumatology* online).

During the follow-up, myocarditis-related complications, such as cardiac death, end-stage heart failure, malignant arrhythmias, or need for an implantable cardioverter defibrillator (ICD), according to current guidelines [21], were recorded.

### Statistical analysis

SPSS22.0 (IBM Corp., Armonk, NY, USA) was employed in analysing the data. Non-parametric tests were employed in the statistical analysis. Categorical variables were analysed using the Fisher’s exact test, and continuous variables were analysed using the Mann–Whitney *U* test. Continuous variables were reported as median and interquartile (IQR) range (25th–75th percentiles), while categorical variables were expressed as numbers and percentages. Spearman’s rank correlation was utilized to correlate the various disease parameters. Univariable and multivariable logistic regression analyses [expressed in terms of odds ratio (OR) and 95% CI] were applied to analyse the predictive ability of baseline variables for myocardial involvement. A *P*-value of <0.05 was accepted as indicating statistical significance.

## Results

### Patient demographics and clinical characteristics

Overall, 43 AAS patients were identified (females 74.4%, median age 58 [48.0–66.0] years). Their demographic, clinical and laboratory characteristics are reported in Table 1.

**Table 1.** Demographic and clinical characteristics of patients with ASS

	Patients with ASS (*n* = 43)
Age (years), median [IQR]	58 [48–66]
Females, *n* (%)	32 (74.4)
Disease duration (months), median [IQR]	6 [4–13]
Autoantibodies	
ANA positivity, *n* (%)	28 (65.2)
Anti-Jo1, *n* (%)	23 (53.5)
Anti-PL-7, *n* (%)	7 (16.3)
Anti-PL-12, *n* (%)	6 (14.0)
Anti-EJ, *n* (%)	3 (7.0)
Anti-SSA, *n* (%)	20 (46.5)
Anti-Ro52, *n* (%)	9 (20.9)
Anti-Ro60, *n* (%)	4 (9.3)
Anti-SSB, *n* (%)	1 (2.3)
Clinical features	
Overall skeletal involvement^a^, *n* (%)	43 (100)
Interstitial lung disease^b^, *n* (%)	31 (72.1)
Myositis^c^, *n* (%)	23 (53.5)
Muscle weakness, *n* (%)	23 (53.5)
Dyspnea, *n* (%)	21(48.8)
Arthralgias, *n* (%)	16 (37.2)
Fever, *n* (%)	16 (37.2)
Raynaud phenomenon, *n* (%)	15 (34.9)
Skin rashes, *n* (%)	15 (34.9)
Myocarditis^d^, *n* (%)	13 (30.2)
Myalgias, *n* (%)	13 (30.2)
Arthritis^e^, *n* (%)	13 (30.2)
Scleroderma pattern at NVC, *n* (%)	10 (23.2)
Dysphagia, *n* (%)	9 (20.9)
Mechanic hands, *n* (%)	9 (20.9)
Palpitations, *n* (%)	4 (9.3)
Weight loss, *n* (%)	4 (9.3)
Calcinosis, *n* (%)	1 (2.3)
MMT score, median [IQR]	76 [70–80]
Laboratory features	
Increased CPK, *n* (%)	23 (53.5)
CPK serum level, median [IQR] (UI/L)	283 [85–1668]
CPK serum level in patients with myositis, median [IQR] (UI/L)	1169 [329.75–4506.75]
LDH serum level (mU/ml), median [IQR]	280.5 [244.25–584]
Increased aldolase, *n* (%)	13 (30.2)
Aldolase serum level (UI/L), median [IQR]	7.80 [5.15–34.6]
Aldolase serum level (UI/L), in patients with myositis, median [IQR]	9.80 [7.60–41.0]
Increased hs-TnT, *n* (%)	17 (39.5)
Hs-TnT serum levels, median [IQR] (ng/l)	23.55 [7.95– 172.75]
Hs-TnT serum levels in patients with myocarditis, median [IQR] (ng/l)	88.0 [23.55– 311.5]
Increased NT-proBNP, *n* (%)	15 (34.9)
NT-proBNP serum levels, median [IQR] (pg/ml)	102.0 [42.0– 407.0]
NT-proBNP serum levels in patients with myocarditis, median [IQR] (pg/ml)	525.5 [243.5– 1575.25]
Increased CPR, *n* (%)	12 (27.9)
Increased ESR, *n* (%)	15 (34.9)
CRP serum levels, median [IQR] (mg/l)	2.0 [1.0–8.5]
ESR serum levels, median [IQR] (mm/1h)	26 [8.0–39.5]

a Overall skeletal involvement includes: myositis, arthritis, arthralgias, mechanic hands.

b Interstitial lung disease evaluated by pulmonary function tests and confirmed by high-resolution CT.

c Myositis diagnosed in the presence of clinical symptoms associated with increase total CPK and/or aldolase plus at least one abnormality at electromyography and/or magnetic resonance.

d Myocarditis considered as specified in the Methods section.

e Arthritis defined as clinical evidence of swollen and tender joints. MMT: Manual Muscle Testing; CPK: creatine phosphokinase; LDH: lactate dehydrogenase; hs-TnT: high-sensitivity troponin T; ASS: anti-synthetase syndrome; *n*: number.

The anti-Jo1 was the most commonly found disease-specific antibody. It was positive in 23 cases (53.5%), followed by anti-PL7 in 7 (16.3%). Anti-PL-12 and anti-EJ were more rarely found (14% and 7%, respectively). Anti-SSA antibody positivity was found in 20 patients (46.5%), together with other disease-specific antibodies in 16 cases.

Muskoloskeletal involvement refers to any signs/symptom related to ASS, as inflammatory arthralgias, arthritis and/or mechanic hands. Myositis was diagnosed in 23 cases (53.5%) and confirmed by muscle biopsy in 10 cases (23.3%). In the remaining 13 cases, the diagnosis of myositis was confirmed by means of electromyography and/or proximal muscle MRI. In 72.1% of the patients, ILD was found through high-resolution chest CT. At baseline, almost half of the patients complained of some degree of dyspnea (48.8%), while palpitations were present in only 4 patients (9.3%). At ASS clinical onset, fever was recorded in 16 patients (37.2%).

Total creatine phosphokinase (CPK) serum levels were increased in 23 patients (53.5%), and aldolase in 13 patients (30.2%). Increased ESR and CRP were observed in 15 (34.9%) and 12 patients (27.9%), respectively. Elevated hs-TnT and NT-proBNP serum levels were found in 17 (39.5%) and 15 patients (34.9%), respectively.

### Therapies

At the time of the first evaluation, the majority of the patients were on steroids (86.0%), at a median (IQR) dose of 5.0 (5.0–15.0) mg of prednisone (or equivalent), for the treatment of musculoskeletal involvement. Overall, based on retrospective records from the entire cohort, the most commonly used immunosuppressant was MMF [26 patients (60.5%)], followed by MTX in 11 cases (25.6%). Concomitant therapy with rituximab, IVIGs or anakinra was prescribed in 15 (34.9%), 6 (13.9%) and 3 patients (7.0%), respectively. Only 7 patients (16.3%) had been previously treated—before ASS with myocarditis diagnosis—with i.v. CYC.

### Patients with ASS diagnosed with myocarditis: clinical features

Among our cohort of 43 patients with ASS, 13 patients (30%) with myocarditis were identified. The diagnosis was made at ASS clinical onset or within the first 3 months of ASS diagnosis in 7 out of the 13 patients (54%). In the remaining cases, myocarditis was diagnosed after a median (IQR) time of 18 (10.5–29.25) months.

In all cases, myocarditis was diagnosed as defined in the methods section. In 5 patients (38.5%), an EMB was performed, including in 2 patients without available CMRI: in all biopsied patients, a virus-negative myocarditis was confirmed.

Clinical, laboratory and instrumental data for patients with ASS diagnosed with myocarditis are reported in Table 2.

**Table 2.** Clinical and instrumental features of patients with ASS with myocarditis

	Patients with ASS with myocarditis (*n* = 13)
Age, median [IQR]	58 [54.25–64.25]
Females, *n* (%)	6 (46.1)
Clinical and biochemical findings	
Subclinical onset, *n* (%)	7 (53.8)
Infarct-like onset, *n* (%)	2 (15.4)
Arrhythmic onset, *n* (%)	2 (15.4)
Congestive heart failure onset, *n* (%)	2 (15.4)
Concomitant myositis, *n* (%)	8 (61.5)
Increased hs-TnT, *n* (%)	12 (92.3)
Hs-TnT serum levels, median [IQR] (ng/l)	88.0 [23.55–311.5]
Increased NT-proBNP, *n* (%)	11 (84.6)
NT-proBNP serum levels, median [IQR] (pg/ml)	525.5 [243.5–1575.25]
Increased CRP, *n* (%)	7 (53.8)
Increased ESR, *n* (%)	7 (53.8)
CRP serum levels, median [IQR] (mg/l)	7.0 [1.7–15.75]
ESR serum levels, median [IQR] (mm/1h)	32.0 [8.0–45.0]
Echocardiogaphy	
Echocardiogaphic abnormalities, *n* (%)	7 (53.8)
Reduced LVEF, *n* (%)	2 (15.4)
LVEF (%), median [IQR]	58.0 [55.0–60.0]
Hypokinesia, *n* (%)	3 (23)
Diastolic dysfunction, *n* (%)	4 (31)
24-h ECG holter analysis	
24-h ECG holter abnormalities, *n* (%)	2 (15.4)
Frequent VEBs, *n* (%)	4 (31)
VEBs number, median [IQR]	1027 [123–1782]
CMRI findings	**11 patients**
Any abnormality, *n* (%)	11 (100)
LGE areas, *n* (%)	10 (91.0)
LGE burden (%), median [IQR]^a^	2.50 [1.75–3.50]
Positive LGE segments (*n*), median [IQR]	2 [1-3]
Pericardial effusion, *n* (%)	9 (81.1)
Reduced LVEF, *n* (%)	4 (36.4)
LVEF (%), median [IQR]	58.0 [51.25–64.25]
EDV-LV (ml), median [IQR]	123.25 [105.30–141.10]
EDV-RV (ml), median [IQR]	137.40 [105.15–150.10]
RVEF (%), median [IQR]	62.0 [56.5–71.0]
Patients with positive T2 ratio on STIR, *n* (%)	6 (54.5)
Mean T2 ratio, median [IQR]	1.90 [1.70–1.95]
Global native T1 relaxation time (ms), median [IQR]	1071 [1042.0–1084.0]
Increased native T1 mapping (ms) (normal value ≤1045 ms), *n* (%)	10 (91.0)
ECV (%; global), median [IQR]	30.5 [27.5–31.0]
Increased ECV (normal value ≤27%), *n* (%)	11 (100)
Increased T2 mapping (normal value ≤50 ms), *n* (%)	10 (91.0)
Global T2 relaxation time (ms), median [IQR]	52.7 [51.25–57.20]
Global T2 relaxation time in patients with increased T2 relaxation time (ms), median [IQR]	53.2 [52.0–58.5]
Lake Louise criteria	
2009 LLC	
Patients with positive regional or global STIR *n* (%)	6 (54.5)
Patients with positive EGE, *n* (%)	0 0
Patients with positive LGE, *n* (%)	10 (91.0)
2009 LLC criteria satisfied^b^, *n* (%)	6 (54.5)
2018 LLC	
Patients with positive T1 criteria, *n* (%)	10 (91.0)
Patients with positive T2 criteria, *n* (%)	10 (91.0)
2018 LLC criteria satisfied, *n* (%)^b^	10 (91.0)

a LGE burden evaluated using the ±3 S.D. method.

b LLC suggestive for active myocardial inflammation. *n*: number; ASS: anti-synthetase syndrome; hs-TnT: high-sensitivity troponin T; LV-EF: left-ventricular ejection fraction; VEBs: ventricular ectopic beats (frequent if higher than 720/24 h); NS-VT: non-sustained ventricular tachycardia; CMRI: cardiac MRI; LV-EDV: left-ventricular end diastolic volume; RV-EDV: right-ventriclular end diastolic volume; BSA: body surface area; ECV: extracellular volume; RV-EF: right-ventricular ejection fraction; STIR: Short-Tau Inversion Recovery; LLC: Lake Louise criteria; EGE: early gadolinium enhancement.

The most common clinical symptom in patients with myocarditis was dyspnea, which was reported by 7 patients (54.4%), while palpitations were reported by 4 patients. All but one patient (92.3%) had increased hs-TnT levels, and 11 patients (84.6%) had increased NT-proBNP serum levels.

Subclinical onset was the most common myocarditis clinical presentation (53.8%). In 2 patients each (15.4% each), myocarditis onset was classified as ‘infarct-like’, ‘arrhythmic’ or ‘congestive heart failure’ (see Supplementary Material, available at *Rheumatology* online). A concomitant skeletal myositis was disclosed in 8 of these cases (61.5%).

In analysing our cohort of patients with ASS with clinically diagnosed myocarditis, 11 patients (91%) underwent CMRI, and in all cases, a diagnosis of myocarditis was confirmed by the presence of at least one CMRI abnormality. In particular, the detected abnormalities in the context of a clinically suspected myocarditis, were: LGE areas, T2 areas of myocardial oedema on STIR images, increased T1 mapping, increased T2 mapping, increased ECV or increased T2 ratio. In the remaining 2 patients who did not perform CMRI, myocarditis was confirmed by EMB. The most common CMRI abnormalities were: increased ECV, present in all cases, areas of LGE (91%), and increased T1 mapping (91%) and T2 mapping (91%). STIR images suggestive of myocardial oedema were detected in 6 patients (54.5%). A concomitant pericardial effusion was present in 9 cases (81.1%), and only 4 patients (36.4%) had a depressed left-ventricular ejection fraction (LV-EF).

**Figure 1.**
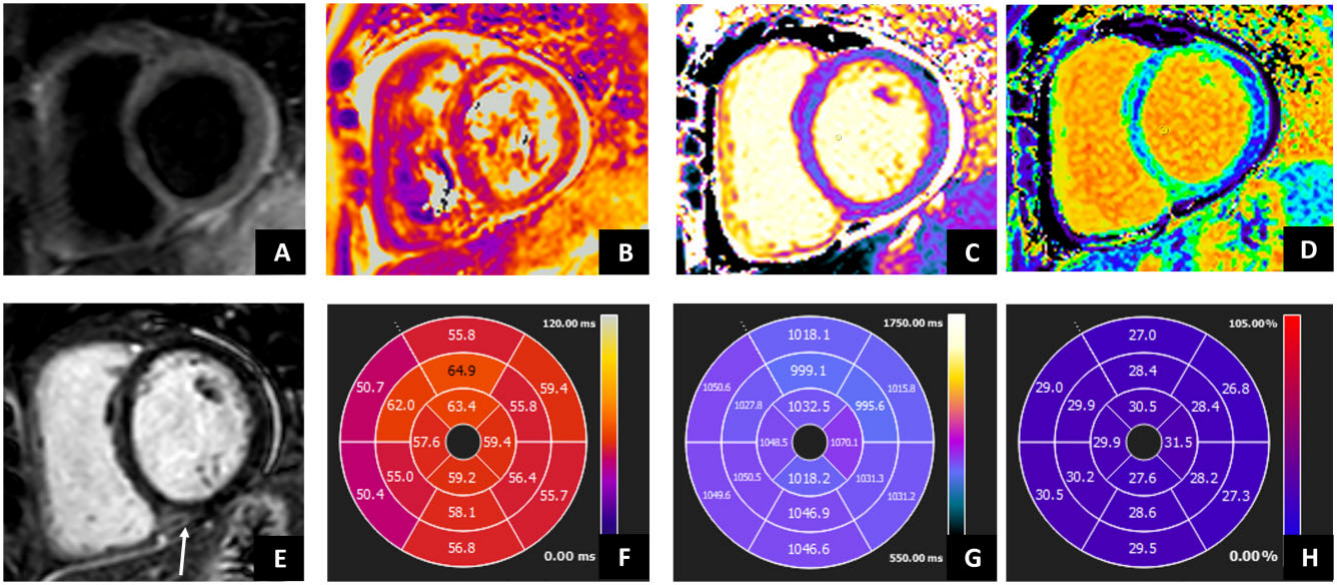
Cardiac MRI in patient with anti-synthetase syndrome. Cardiac MRI shows ventricles with normal volumes and function. STIR images showed (A) absent focal oedema, but subtle diffuse oedema as showed by (B) T2 mapping and (F) AHA bullseye (global value 58 ms; normal value ≤50 ms). (E) Late gadolinium enhancement showed mid-wall hyperintensity in the basal posterior septum, associated with normal native T1 mapping (C and G) (1037 ms; normal value <1045 ms) but increased ECV values (D and H) (29%, normal values <27%), as for mild increase in interstitial fibrosis. Moreover, mild pericardial effusion was evident without significant increase in the pericardium thickness. AHA: American Heart Association; STIR: Short-tau Inversion Recovery; ECV: extracellular volume

The 2009 Lake Louise criteria (LLC) were satisfied by 6 patients, whereas the 2018 LLC were satisfied by 10 patients. Thus, with the updated CMRI criteria for myocarditis diagnosis, which include T1 mapping and T2 mapping, the sensitivity improved from 54.6% to 91.0% (Fig. 1).

In patients with ASS diagnosed with myocarditis who underwent CMRI, no statistically significant correlation emerged between CMRI parameters (LVEF, T2 ratio, T1 mapping, T2 mapping, and ECV) and disease parameters (hs-TnT, NTproBNP, ESR, CRP and CPK serum levels) (*P*-value non-significant).

At the time of myocarditis diagnosis, 11 out of 13 patients (84.6%) were on steroids, at a median (IQR) dose of 5.0 (7.5–18.75) mg of prednisone (or equivalent). Only 5 out of 13 patients (38.5%) were already being treated with immunosuppressants, mainly for ILD, arthritis and/or myositis. Specifically, 2 patients were on MTX, 2 patients with severe disease complicated by myositis and ILD received i.v. CYC (combined with IVIG in one case), and 1 patient was on AZA.

Once myocarditis was diagnosed, all patients were treated with steroids 1 mg/kg per day (then tapered within 4 months), preceded by i.v. methylprednisolone (1 g for 3 consecutive days) in 6 patients (46.1%) with concomitant severe myositis.

Twelve patients (92.3%) were treated with a combination of CSs and MMF [up to 1500 mg per day in 3 patients (25%), up to 2 g per day in 4 patients (33.3%), and up to 3 g per day 5 patients (41.7%)]. The remaining patient (7.7%) received AZA. In 3 cases (23.1%) with severe or refractory disease, rituximab and anakinra were administered as additional second-line therapy.

During follow-up, 3 patients presented a myocarditis relapse, which was managed with an increase in the CS dose combined with anakinra in 1 case and with rituximab in 2 patients with concomitant myositis relapse. One patient (7.7%) with myocarditis and signs and symptoms of inflammation (high CRP and fever), already on steroids, MMF and anakinra, died of sudden cardiac death upon anakinra suspension. Two (15.4%) patients required ICD insertion for arrhythmic complications.

### Clinical features of patients with ASS with and without myocarditis

To identify the clinical features of patients with ASS that were associated with myocardial involvement, clinical, immunological, laboratory and instrumental characteristics of patients with ASS with myocarditis were compared with those of patients with ASS without myocarditis.

Laboratory data for the patients with myocarditis were collected at clinical onset, before the initiation of specific immunosuppressive therapy. The laboratory data for the patients without myocarditis were obtained at the first clinical evaluation.

Patients with ASS with myocarditis were more frequently males (53%) compared with those without myocarditis (13%) (*P* = 0.009), while age and disease duration were similar when comparing patients with ASS with and without myocarditis (*P*-value non-significant for both comparisons). No significant differences emerged when comparing the presence of traditional cardiovascular risk factors or autoantibody profiles in patients with ASS with and without myocarditis.

As expected, patients with ASS with myocarditis had higher levels of hs-TnT [88.0 (23.55–311.5) vs 9.80 (5.0–23.0) ng/l] and NTproBNP [525.5 (243.5–1575.25) vs 59.0 (32.0–165.5) pg/ml] (*P* < 0.001 and P = 0.013, respectively), while CPK levels did not differ between groups (*P*-value non-significant). Liver enzymes (AST and ALT) and CRP were markedly increased in ASS patients with myocarditis compared with those without myocarditis [97.5 (40.0–194.0) vs 27.5 (21.0–34.25) U/l, 136.5 (32.25–194.0) vs 212.5 (12.0–33.25) U/l and 7.0 (1.7–15.75) vs 1.85 (0.5–2.86) mg/l, respectively (*P* = 0.007, *P* = 0.006 and *P* = 0.011, respectively).

No statically significant differences emerged for dyspnea, muscle involvement, ILD, or arthritis between the two groups of patients, while fever at ASS presentation was more frequently observed in patients with ASS with a diagnosis of myocarditis (69% *vs* 17%, *P* = 0.001). No significant differences in terms of echocardiographic abnormalities between the two groups emerged. At multivariate logistic regression analysis, only the presence of fever at ASS clinical onset (OR 7.707, 95% CI 1.026–57.917, *P* = 0.047) was associated with myocarditis (Table 3).

**Table 3.** Clinical features of patients with ASS with and without myocarditis

	Patients with ASS with myocarditis (*n* = 13)	Patients with ASS without myocarditis (*n* = 30)	*P*
Age, median [IQR]	58 [54.25–64-25]		0.641
Females, *n* (%)	6 (46)	26 (87)	**0.009**
CV risk factors			
Smoking, *n* (%)	6 (46)	7 (23)	0.173
Arterial hypertension, *n* (%)	4 (31)	11 (37)	0.739
Obesity, *n* (%)	2 (15)	2 (7)	0.589
Diabetes, *n* (%)	1 (8)	2 (7)	0.999
Ischemic heart disease, *n* (%)	2 (15)	3 (10)	0.637
Autoantibodies			
Anti-Jo1, *n* (%)	7 (54)	16 (53)	0.999
Anti-PL7, *n* (%)	3 (23)	4 (13)	0.655
Anti-PL12, *n* (%)	1 (8)	5 (17)	0.649
Anti-EJ, *n* (%)	1 (8)	2 (7)	0.999
Anti-SSA, *n* (%)	7 (54)	13 (43)	0.740
Clinical features			
Fever, *n* (%)	9 (69)	5 (17)	**0.001**
Muscle weakness, *n* (%)	8 (61)	15 (50)	0.526
Myalgias, *n* (%)	6 (46)	7 (23)	0.163
RP, *n* (%)	5 (38)	10 (33)	0.742
ILD, *n* (%)	10 (77)	21 (70)	0.727
Dyspnea, *n* (%)	7 (54)	14 (47)	0.747
Dysphagia, *n* (%)	3 (23)	6 (20)	0.999
Mechanical hands, *n* (%)	3 (23)	6 (20)	0.999
Arthritis, *n* (%)	5 (38)	8 (27)	0.485
MMT score, median [IQR]	78.0 [73.0–80.0]	75.5 [70.0–80.0]	0.564
Biochemistry			
CPK serum levels, median [IQR] (UI/l)	337.0 [110.0–3959.75]	243.0 [80.25–1268.0]	0.249
LDH serum levels, median [IQR] (UI/l)	609.0 [267.0–747.75]	265.5 [251.0–392.75]	0.249
CRP serum levels, median [IQR] (mg/l)	7.0 [1.7–15.75]	1.85 [0.5–2.86]	**0.011**
ESR, median [IQR] (mm/h)	32.0 [8.0–45.0]	23.0 [8.75–37.0]	0.584
AST (U/l), median [IQR]	97.5 [40.0–194.0]	27.5 [21.0–34.25]	**0.007**
ALT (U/l), median [IQR]	136.5 [32.25–194.0]	212.5 [12.0–33.25]	**0.006**
Cardiac enzymes			
Hs-TnT serum levels, median [IQR] (ng/l)	88.0 [23.55–311.5]	9.80 [5.0–23.0]	**<0.001**
NT-proBNP serum levels, median [IQR] (pg/ml)	525.5 [243.5–1575.25]	59.0 [32.0–165.5]	**0.013**
Echocardiogaphy			
LV-EF (%), median [IQR]	58.0 [55.0–60.0]	63.5 [60.0–65.0]	0.076
LV-EF reduced, *n* (%)	2 (15)	2 (9)	0.586
Hypokinesia, *n* (%)	3 (23)	1 (4)	0.106
Diastolic dysfunction, *n* (%)	4 (31)	2 (9)	0.148

ASS: anti-synthetase syndrome; *n*: number; CV: cardiovascular; ILD: interstitial lung disease; MMT: Manual Muscle Testing; CPK: creatine phosphokinase; LDH: lactate dehydrogenase; hs-TnT: high-sensitivity troponin T; AST:aspartate aminotransferase; ALT: alanine aminotransferase; LV-EF: left-ventricular ejection fraction.

Bold typeface refers to *P* values that are statistically significant.

## Discussion

Our study shows that myocarditis is a frequent and likely overlooked manifestation in patients with ASS and that it can be one of the presenting, even isolated, features associated with ASS. We also highlighted that CMRI mapping techniques are fundamental to improving the sensitivity to detect myocardial inflammation in patients with ASS. Moreover, among disease features, the presence of fever and raised CRP were associated in our cohort with myocardial involvement at disease onset.

In contrast to previous studies that reported a low prevalence of myocarditis in patients with ASS, we observed a significant frequency of myocarditis in our cohort (30%).

A large retrospective series of patients with ASS (a cohort of 300 patients belonging to the French National Registry) reported a prevalence of myocarditis of 3.4%, as only 12 cases of myocarditis were identified from 2000 to 2014 [2]. Similarly, within the Johns Hopkins Myositis Center Research Registry, of 3082 adult patients with IIM, 14 patients were identified as having myocarditis, when applying an encounter code of myocarditis from 2004 to 2021 [22].

In our cohort, the significantly high prevalence of myocarditis may be due to several reasons. First, our centre is a tertiary regional referral centre for myocarditis, and a Myocarditis Disease Unit is operating at our centre. Therefore, an over-referral of patients with ASS with suspected myocarditis may be expected. Second, our standard of care includes a thorough myocardial evaluation for all patients with CTDs. This includes evaluation of signs and symptoms suggestive for cardiac involvement, assessment of cardiac enzymes, standard 12-leads ECG, echocardiography and monitoring with a 24-h ECG holter for all patients. CMRI is also performed in all patients with suspicion of myocardial involvement. Thus, early and/or subclinical myocarditis could be identified in most cases. Finally, the more extensive use of CMRI as a non-invasive diagnostic tool for detecting myocarditis, as well as the inclusion of novel CMRI techniques from 2000–2014 vs 2016–2022, may explain the higher prevalence of myocarditis in our recent ASS cohort.

Importantly, myocarditis was diagnosed at the clinical onset of ASS in >50% of patients, thus representing a potential early manifestation of the disease. This is in agreement with the results from the French cohort, in whom myocarditis was the first disease manifestation in 42% of cases [2].

Diagnosing myocarditis remains a challenge in patients with autoimmune diseases, however, since clinical signs could be absent or not specific, or concealed by other clinical features. This suggests that a comprehensive evaluation of cardiac involvement is needed in all patients with ASS. Indeed, myocarditis was rarely detectable by echocardiography. Echocardiographic abnormalities suggestive for myocardial involvement were only apparent in approximately half of the patients with ASS who were eventually diagnosed by CMRI. Importantly, a decreased LV-EF was only rarely detected and, consistently, clinical onset with heart failure was not recorded in our cohort. In the French cohort, left or right ventricular dysfunction at echocardiography was present in the vast majority of myocarditis patients, and these findings were paralleled by the presence of congestive heart failure in 75% of cases, leading to admission to the intensive care unit in 50% of cases [2]. Similarly, in the study by Chung, the vast majority of patients had a clinically severe myocarditis, requiring hospitalization in 93% of cases due to depressed LV-EF in 71% of cases [22]. These results underline again the different clinical profiles of the patients with ASS with myocarditis in the previous studies compared with the clinical profiles of our patients with ASS with myocarditis. This discrepancy is at least partially due to the method used for identifying myocardial involvement. In both the French and American cohorts, patients were retrospectively identified from registries, whereas in our study we systematically evaluated the presence of myocardial involvement in all patients with ASS. As a consequence, an earlier diagnosis of even subclinical myocarditis was possible in our cohort. Unfortunately, though, while EMB is the diagnostic gold standard for diagnosing myocarditis, the limited sensitivity and the risks associated with the procedure limit its regular use. For this reason, CMRI is an alternative non-invasive diagnostic tool for myocarditis [7–10, 23]. In CTDs, CMRI has been widely used to evaluate cardiac involvement and has progressively become a key diagnostic and prognostic tool [5–12, 15, 23–28]. Historically, the CMRI diagnosis of myocarditis relies on the LLC [7], which include only non-parametric techniques depending on a qualitative or semi-quantitative analysis of signal intensities on STIR, EGE and LGE images. In suspected acute myocarditis, the CMRI with the LLC has a diagnostic accuracy close to 80%. However, these criteria have important limitations when applied to patients with CTDs [12]. STIR images have a modest sensitivity for detecting diffuse myocardial oedema, and this is further reduced by the need for muscle signal intensity as reference tissue, with subsequent risk of false-negative results in patients with coexistent skeletal myositis [12].

T1 mapping, T2 mapping, and ECV have emerged as novel techniques that can overcome these limitations [8, 23, 24]. In particular, T1 and T2 mapping are more sensitive than STIR in detecting diffuse myocardial oedema [7, 23, 24]. This was also shown in our cohort, in whom CMRI sensitivity for diagnosing myocarditis improved with the inclusion of mapping techniques, specifically T2 mapping, based on the updated LLC. When the native T1 mapping, T2 mapping, and ECV were included in the 2018 revised LLC [8] for our patients, the sensitivity of CMRI increased from 54.6% to 91.0%. This improvement was mainly driven by the additional value of T2 mapping in detecting subtle oedema. This notion is of great clinical importance, since the early identification of myocarditis is mandatory to establishing prompt therapeutic intervention and improving the outcomes for patients. The timely use of immunosuppressive drugs may be a successful approach in ASS with myocarditis [2], and, in particular in the early phases of the disease, the treatment can prevent the progression to late-stage cardiac damage.

In our cohort, myocarditis was more common in males and clinically manifested with dyspnea in almost all cases, or palpitations, which were reported by a minority of patients. These non-specific symptoms were almost always associated with increased troponin serum levels, which were elevated in all but one patient, whereas NT-proBNP was raised in 85% of cases. Therefore, cardiac enzymes should be routinely measured in all patients with ASS, either at baseline or during follow-up.

The greater occurrence of myocarditis in males is in keeping with previous studies reporting a sex ratio for myocarditis of 1:2–4 females to males [29]. Toll-like receptor (TLR)2 and TLR4 signalling pathways, which are elevated in male individuals, are considered responsible for this imbalance, as they play a central role in increasing inflammation during myocarditis and in promoting remodelling and fibrosis [29].

The finding that a significantly higher percentage of patients with ASS with myocarditis, compared with those without myocarditis, had fever and increased CRP levels at ASS clinical onset suggests that an inflammatory phenotype does exist and that myocarditis is one of the primary clinical features. In ASS, the presence of an inflammatory phenotype and its association with a more aggressive disease has already been reported in a large number of Chinese patients [30]. Based on these findings, a high suspicion of myocarditis should always be borne in mind when dealing with patients with ASS with such inflammatory features. Of note, in our cohort, two patients were fully responsive to the IL-1 blocking agent anakinra. Consistently, the efficacy of anakinra in treating both patients with refractory ASS and those with different types of myocarditis has been recently reported [31–35]. This evidence may indicate a possible pathogenic role of IL-1–mediated inflammation in ASS with myocarditis.

Our data show that myocarditis in patients with ASS could be diagnosed using a comprehensive algorithm that includes analysis of clinical signs and disease characteristics, cardiac biomarkers, and instrumental evaluation, including CMRI with mapping techniques.

To the best of our knowledge, this is the first study that has comprehensively evaluated the presence and the clinical and instrumental features of myocarditis in patients with ASS. Similarly, we assessed for the first time the additional value of CMRI mapping techniques in the detection of myocardial inflammation in patients with ASS with suspected myocarditis. Refining the diagnostic approach for detecting myocardial inflammation, taking into account the phenotype of patients with ASS with myocarditis, and by using CMRI with mapping techniques could pave the way to novel and earlier therapeutic perspectives.

An additional strength of our study is the comprehensive characterization of ASS with myocarditis in all patients at a dedicated multidisciplinary Myocarditis Disease Unit, using a rigorous algorithm.

Nevertheless, our study has some limitations. The high rate of myocarditis we observed was at least partially due to the routine assessment of myocardial involvement in a dedicated Myocarditis Disease Unit with readily available CMRI. The differences found between patients with and without myocarditis were determined after the diagnosis of myocarditis (and most of them are part of the diagnostic criteria used to define myocarditis, as cardiac biomarkers). As a consequence, even considering the finding at multivariate analysis of fever at ASS diagnosis as associated with the occurrence of myocarditis, our results need to be confirmed. The low number of patients may be considered a limitation for drawing solid conclusions, even though the rarity of the disease (and of this specific complication) needs to be taken into account. Moreover, EMB was performed in only five patients, thus precluding the possibility of correlating histological features with CMRI parameters. The consensus reading of CMRI represents a methodological limitation. To date, the segmentation is performed using advanced dedicated cardiac MRI software, which allows automatic segmentation based on artificial intelligence algorithms, further checked and manually corrected by an experienced operator. Moreover, the qualitative assessment of the signal intensities was supported by semi-quantitative analysis performed automatically on the same software, thus limiting subjectivity in the image interpretation. Finally, due to the retrospective nature of our study, we could not assess the prognostic role of the clinical and CMRI features in ASS with myocarditis.

## Conclusions

Myocarditis is frequent in patients with ASS, especially in males, and could be one of the presenting features of the disease. Patients with ASS with myocarditis more frequently had fever and increased inflammatory markers compared with those without myocarditis, suggesting that a more inflammatory phenotype may be associated with myocarditis. Novel CMRI mapping techniques with the inclusion of T2 mapping increase the diagnostic sensitivity for ASS-myocarditis.

## Supplementary Material

kead541_Supplementary_Data

## Data Availability

The data underlying this article will be shared on reasonable request to the corresponding author.
