# Supplementary material for: Myocarditis in anti-synthetase syndrome: clinical features and diagnostic modalities
Source: Rheumatology (Oxford). 2023 Oct 5;63(7):1902–10. doi: 10.1093/rheumatology/kead541 (PMC11215987; doi:10.1093/rheumatology/kead541)
Supplement: kead541_Supplementary_Data [file kead541_supplementary_data.docx]

**Supplementary Material**

***Patients and Methods***

All patients were followed-up at the Inflammatory Myopathies Outpatients Clinic and Myocarditis Disease Unit of IRCCS San Raffaele Hospital.

Myocarditis Disease Unit is a tertiary multidisciplinary referral Unit for inflammatory cardiomyopathies which includes rheumatologists, immunologists, cardiologists, and radiologists.

All ASS patients underwent the same approach, which included cardiac enzymes, standard 12-leads ECG, 24-hour ECG-Holter and echocardiography at baseline. In those with suspected myocarditis [presence of symptoms and/or signs of cardiac involvement (dyspnea, palpitations, chest pain, signs of congestive heart failure) associated with increased serum levels of high-sensitive troponin T(hs-TnT) and/or NT-proBNP] CMR is requested.

In those with myocarditis the same evaluations are also performed at 3, 6, and 12 months after the diagnosis of myocarditis. Thereafter, the same procedures are repeated when clinically needed during follow-up.

Traditional cardiovascular risk factors were recorded in all patients, and included: current or past smoking history, familiar history for cardiovascular disease, overweigh or obesity based on body mass index, diabetes mellitus, dyslipidemia, systemic arterial hypertension.

***Myocarditis onset: clinical presentation pattern***

The predominant signs or symptoms that led to the suspicion of myocarditis were considered for the definition of the clinical presentation. Dyspnea, atypical chest pain and palpitations were considered ‘symptoms’, while syncope and lower limb edema were considered ‘signs’ of possible myocarditis.

According to the New York Heart Association (NYHA), classification criteria dyspnea was categorized into four classes[14].

The onset of ASS-related myocarditis was considered as “arrhythmic” when ventricular life-threatening arrhythmic complications were the presenting sign (i.e. non-fatal episodes of cardiac arrest, frequent ventricular ectopic beats[VEBs], non-sustained ventricular tachycardia[NS-VT]); as “infarct-like” in case of chest pain associated with markedly increased hs-cTnT; as “heart failure” in case of clinical signs and symptoms of congestive heart failure; and as “subclinical” when there was a persistent, even if modest, increase of cardiac biomarkers in a pauci-symptomatic patient, as previously reported [2,3].

***Cardiac magnetic resonance***

T2-STIR was obtained with a body coil. T2-Weighted Short-tau Inversion Recovery (STIR) edema-sensitive images were obtained in the short axes view from base to apex and in the 2-chambers and 4- chambers long axis planes (repetition time [TR]=1860 ms; echo time [TE]=100 ms; flip angle=90°; slice thickness=12mm, gap=0) [4].

For volumes and function analysis cine steady-state free precession(cine-SSFP) images were obtained in four-, two- and three-chamber long axis and in two-chambers short-axis (repetition time [TR] = 3.4 ms; echo time [TE]=1.7 ms; flip angle = 60°; slice thickness=8 mm, gap=0, 30 cardiac phases per heartbeat) [4].

LGE imaging was performed by employing a 2D inversion recovery gradient-echo sequence with inversion time optimized to null the signal of normal myocardium (slice thickness=8 mm, slice gap=0 mm, voxel size=1.6×1.8 mm, reconstruction matrix=384), after at least 5 minutes after the rest perfusion.

T2-mapping, native, and 15 minutes post-contrast T1-mapping were obtained in 3 subsequent matched short-axis slices which are base, mid-ventricle, and apex) [4].

T2-mapping was performed by employing a gradient-(echo planar imaging) and spin-echo multi-echo sequence (GraSE) and T1-mapping using modified Look-Locker inversion recovery (MOLLI) sequence with 5(3)3 and 4(1)3(1)2 sampling scheme for native and post-contrast, respectively(90).

Gadolinium [0.15mmol/kg of Gadobutrol (Gadovist, Bayer Healthcare, Berlin, Germany)] was injected automatically from an antecubital vein of the right arm at 3.5ml/sec, followed by a saline flush (20ml at 3.5ml/sec) [4].

All CMR examinations were centrally analyzed using dedicated software (CVI42v.5.6.6, Circle Cardiovascular Imaging, Calgary, Canada), with an agreement between two skilled observers (A.P. and E.A., with 12 and 20 years of experience, respectively), who were blinded to the clinical presentation and results.

After drawing the endocardial and epicardial borders in the end-diastolic and end-systolic short-axis cine-SSFP images, the LV end-diastolic volume (LVEDV), ejection fraction (LVEF), and mass (EDWM) were obtained automatically.

Areas of myocardial hyperintensity with a non-ischemic pattern were used to assess myocardial edema qualitatively, and a ratio between myocardial and skeletal muscle signal intensity (SI) (T2-ratio, positive 1.9) was used to assess myocardial edema semi-quantitatively [4,5].

LGE was assessed in terms of the visual number of myocardial segments that are involved, the transmural pattern, and the percentage of the injured myocardium, the latter was obtained by setting a SI threshold of +3SD.

Endocardial and epicardial borders were drawn on the 3 short-axis, which are base, mid-ventricle, apex, T1- and T2-maps. By setting an automatic offset of 10% from endocardial and epicardial borders the partial volume of blood and fat was minimized.

ECV was calculated by this equation: ECV=(1−hematocrit)×[ΔR1myocardium]/[ΔR1blood-pool], where ΔR1 signifies the difference in pre-contrast and post-contrast relaxation rates(1/T1).

***Endomyocardial biopsy***

EMB was performed according to current guidelines based on clinical suspicion of myocarditis, and as previously described [6-13]. Multiple biopsies from the right side of the interventricular septum (five to six samples from each patient) were obtained via the internal jugular vein, using a preformed long sheath and a disposable bioptome (Cordis,Johnson and Johnson,Hialeah, FL,USA). Between three to five samples per patient were processed for histology and immunohistochemistry. For histology, multiple 5 mm-thick sections were cut and stained with haematoxylin– eosin, elastic Van Gieson, Masson’s trichrome and Heidenhain’s trichrome stain, and examined by light microscopy. Diagnosis of myocarditis was based on the Dallas criteria and on lymphocyte and/or macrophage count>14 per mm^2^ [12]. Two frozen myocardial specimens per patients were used to detect genome of cardiotropic virus by polymerase chain reaction. To exclude passive contamination from blood, blood samples were collected for each patient at the same time as EMB and tested for the same virus in the presence of a positive EMB result. To characterize inflammatory infiltrates, immunohistochemistry was performed using antibodies against CD3,CD43,CD45,CD20, and CD68 (Dako Corp.,Glostrup,Denmark). The degree of myocardial fibrosis on EMB was calculated in the most representative heart specimens for each patient and assessed as relative percentage[5].

**Results: ASS patients with subclinical onset of myocarditis.**

Among the 13 ASS patients with myocarditis, 7 were classified as having a subclinical onset, as previously defined. In all but one ASS patients with subclinical onset of myocarditis (6 out of 7), diagnosis of myocarditis was made at ASS clinical onset (or within 3 months); in the remaining patient, the subclinical onset of myocarditis was diagnosed during ASS disease course, and precisely after 18 months from ASS onset; this patient was already treated with prednisone (5 mg daily) and mycophenolate mofetil. Overall, considering the 7 patients with subclinical onset, 6 (85.7%) were already treated with prednisone at time of myocarditis diagnosis (for musculoskeletal involvement), at a median daily dosage of 7.5 [5.0 – 18.75] mg, and 3 (42.8%) were already treated with immunosuppressants.

Patients with subclinical onset of myocarditis had a less inflammatory phenotype characterized by lower serum levels of CRP and lower frequency of fever compared to patients with ASS patients with a different onset of myocarditis. Lower values of ECV% at CMR were found in patients with subclinical onset of myocarditis compared to others. Results are summarized in Supplementary Table S1.

**Supplementary Table S1. Disease features of ASS patients with subclinical or non-subclinical onset of myocarditis.**

|  | **Subclinical onset (n=7)** | **Non-subclinical onset (n=6)** | **p** |
| --- | --- | --- | --- |
| **Fever, n (%)** | 3 (42.8) | 6 (100) | **0.049** |
| **Hs-TnT (ng/L), median [IQR]** | 109.5 [21.0 – 888.25] | 390.0 [93.5 – 1046.0] | 0.940 |
| **NT-proBNP (pg/mL), median [IQR]** | 212.5 [94.25 – 268.5] | 807.0 [135.5 – 7336.5] | 0.058 |
| **CPK (UI/L), median [IQR]** | 982.0 [74.0-5678.0] | 337 [263.5 – 3479.5] | 0.059 |
| **CRP (mg/L), median [IQR]** | 3.35 [1.05 – 25.0] | 6.6 [2.07 – 101.8] | **0.040** |
| **ESR (mm/1h), median [IQR]** | 30.0 [7.5 – 44.5] | 48.0 [7.0 – 53.0] | 0.275 |
| **LV-EF at CMR (%), median [IQR]** | 62.0 [56.5 – 68.5] | 58.0 [50.0 – 67.5] | 0.499 |
| **T1 mapping (ms), median [IQR]** | 1102.5 [1074.25 – 1141.25] | 1039.0 [1009.5 – 1135.25] | 0.291 |
| **T2 mapping (ms), median [IQR]** | 51.5 [49.2 – 57.2] | 55.0 [51.6 – 58.75] | 0.859 |
| **ECV (%), median [IQR]** | 27.5 [26.5 – 37.9] | 30.8 [29.4 – 32.2] | **0.004** |
| **LGE present, n (%)** | 6 (85.7) | 5 (83.3) | 0.731 |
| **Previous therapy*, n (%)** | 4 (57.1) | 2 (33.3) | 0.383 |

*n=number; ASS= anti-synthetase syndrome; hs-TnT=high sensitive troponin T; CRP= C-reactive protein; ESR= erythrocyte sedimentation rate; LV-EF=left-ventricular ejection fraction; CMR=cardiac magnetic resonance; ECV=extra-cellular volume; LGE= late gadolinium enhancement.*

**steroids and/or immunosuppressants*

**References**

1. Harvey RM, Doyle EF, Ellis K. Major changes made by Criteria Committee of the New York Heart Association. Circulation. 1974;49(3):390.
2. Peretto G, Sala S, De Luca G, Campochiaro C, Sartorelli S, Cappelletti AM, et al. Impact of systemic immune-mediated diseases on clinical features and prognosis of patients with biopsy-proved myocarditis. Int J Cardiol. 2019 Apr 1;280:110-6.
3. De Luca G, Palmisano A, Campochiaro C, Vignale D, Cavalli G, Bruno E, Sartorelli S, Ferlito A, Peretto G, Sala S, Matucci-Cerinic M, Dagna L, Esposito A. Cardiac magnetic resonance in systemic sclerosis myocarditis: the value of T2 mapping to detect myocardial inflammation. Rheumatology (Oxford). 2022 Feb 21:keac098. doi: 10.1093/rheumatology/keac098.
4. De Luca G, Palmisano A, Campochiaro C, Vignale D, Cavalli G, Bruno E, et al. Cardiac magnetic resonance in systemic sclerosis myocarditis: the value of T2 mapping to detect myocardial inflammation. Rheumatology. 2022 Nov 2;61(11):4409–19.
5. Friedrich MG, Sechtem U, Schulz-Menger J, Holmvang G, Alakija P, Cooper LT, et al. Cardiovascular Magnetic Resonance in Myocarditis: A JACC White Paper. Vol. 53, Journal of the American College of Cardiology. 2009. p. 1475–87.
6. Peretto G, Sala S, De Luca G, Campochiaro C, Sartorelli S, Cappelletti AM, et al. Impact of systemic immune-mediated diseases on clinical features and prognosis of patients with biopsy-proved myocarditis. Int J Cardiol. 2019 Apr 1;280:110-6.
7. De Luca G, Campochiaro C, De Santis M, Sartorelli S, Peretto G, Sala S, et al. Systemic sclerosis myocarditis has unique clinical, histological and prognostic features: a comparative histological analysis. Rheumatology (Oxford). 2020 Sep 1;59(9):2523-33.
8. Pieroni M, De Santis M, Zizzo G, Bosello S, Smaldone C, Campioni M, et al. Recognizing and treating myocarditis in recent-onset systemic sclerosis heart disease: potential utility of immunosuppressive therapy in cardiac damage progression. Semin Arthritis Rheum 2014;43:526–35.
9. De Luca G, Bosello SL, Gabrielli FA, Berardi G, Parisi F, Rucco M, et al. Prognostic Role of Ventricular Ectopic Beats in Systemic Sclerosis: A Prospective Cohort Study Shows ECG Indexes Predicting the Worse Outcome. PLoS One. 2016 Apr 21;11(4):e0153012.
10. De Luca G, Campochiaro C, Sartorelli S, Franchini S, Candela C, Peretto G, et al. Unexpected acute lymphocytic virus-negative myocarditis in a patient with limited cutaneous systemic sclerosis: a case report. Scand J Rheumatol 2018;30:1–2.
11. Bosello S, De Luca G, Ferraccioli G. Troponin in stable ischemic heart disease and diabetes. N Engl J Med 2015;373:1977–8.
12. De Luca G, Bosello S, Leone AM, Gabrielli F, Pelargonio G, Inzani F, et al. Life-threatening arrhythmias in a scleroderma patient: the role of myocardial inflammation in arrhythmic outburst. Scand J Rheumatol. 2017 Jan;46(1):78-80.
13. Leone O, Veinot JP, Angelini A, Baandrup UT, Basso C, Berry G, et al. 2011 consensus statement on endomyocardial biopsy from the Association for European Cardiovascular Pathology and the Society for Cardiovascular Pathology. Cardiovasc Pathol. 2012;21(4):245-74.
14. Aretz HT. Myocarditis: the Dallas criteria. Hum Pathol. 1987;18(6):619-24.
